# Crystal structure and Hirshfeld surface analysis of 4-[4-(1*H*-benzo[*d*]imidazol-2-yl)phen­oxy]phthalo­nitrile dimethyl sulfoxide monosolvate

**DOI:** 10.1107/S2056989019006510

**Published:** 2019-05-10

**Authors:** Sibel Demir Kanmazalp, Pınar Şen, Necmi Dege, Salih Zeki Yildiz, Namık Ozdemir, Irina A. Golenya

**Affiliations:** a Gaziantep University, Technical Sciences, 27310, Gaziantep, Turkey; b Centre for Nanotechnology Innovation, Department of Chemistry, Rhodes University, Grahamstown, South Africa; c Ondokuz Mayıs University, Faculty of Arts and Sciences, Department of Physics, 55139 Samsun, Turkey; d Sakarya University, Faculty of Arts and Sciences, Department of Chemistry, 54187, Sakarya, Turkey; e Department of Mathematics and Science Education, Faculty of Education, Ondokuz Mayıs University, Samsun, Turkey; f Taras Shevchenko National University of Kyiv, Department of Chemistry, 64, Vladimirska Str., Kiev 01601, Ukraine

**Keywords:** crystal structure, phthalo­nitrile, imidazole, Hirshfeld analysis, hydrogen bonds

## Abstract

This work presents the synthesis and structural characterization of [4-(1*H*-benzo[*d*]imidazol-2-yl)phen­oxy]phthalo­nitrile, a phthalo­nitrile derivative carrying a benzimidazole moiety. The compound crystallizes as its dimethyl sulfoxide monosolvate, C_21_H_12_N_4_O·(CH_3_)_2_SO. The dihedral angle between the two fused rings in the heterocyclic ring system is 2.11 (1)°, while the phenyl ring attached to the imidazole moiety is inclined by 20.7 (1)° to the latter. In the crystal structure, adjacent mol­ecules are connected by pairs of weak inter­molecular C—H⋯N hydrogen bonds into inversion dimers. N—H⋯O and C—H⋯O hydrogen bonds with *R*_2_^1^(7) graph-set motifs are also formed between the organic mol­ecule and the disordered dimethyl sulfoxide solvent [occupancy ratio of 0.623 (5):0.377 (5) for the two sites of the sulfur atom]. Hirshfeld surface analysis and fingerprint plots were used to investigate the inter­molecular inter­actions in the crystalline state.

## Abstract 2

The synthesis, structural characterization and Hirshfeld surface analysis of 4-[4-(1*H*-benzo[*d*]imidazol-2-yl)phen­oxy]phthalo­nitrile, a substituted phthalo­nitrile derivative carrying a benzimidazole functional group, are reported.

## Chemical context   

Benzimidazole and its derivatives are some of the oldest and chemically most-studied nitro­gen-containing aromatic heterocyclic compounds (Srestha *et al.*, 2014 ▸). They have a wide range of applications in medicinal chemistry and in biological processes including as anti­cancer, anti­ulcer, anti­fungal and anti-inflammatory agents, and exhibit anti­mycobacterial and anti­oxidant activities (El Rashedy & Aboul-Enein, 2013 ▸; Gaba *et al.*, 2014 ▸; Kathiravan *et al.*, 2012 ▸). They are also used as ligands with fluorescent properties. The fluorescent characteristic of these compounds can be changed by substitution or derivatization of different groups at the NH position of the benzimidazole skeleton.

Phthalo­nitrile derivatives are some of the most widely used precursors for the preparation of phthalocyanines (Pc). The preparation of phthalocyanines is frequently carried out by a cyclo­tetra­merization reaction of phthalo­nitriles. The synthesis of the latter compound family, carrying different functional groups, leads to functionalized phthalocyanines that are of great importance with respect to new mol­ecular materials and targeted applications such as catalysis, liquid crystals, photosensitizers for photodynamic therapy (PDT), non-linear optics, nanotechnology or dye-sensitized solar cells (Torre *et al.*, 2004 ▸; Martínez-Díaz *et al.*, 2011 ▸). In this context, we have recently described a model study, *i.e.* the synthesis, characterization and Hirshfeld surface analysis of zinc phthalocyanines carrying benzimidazole groups through oxygen bridges to a Zn–Pc core (Sen *et al.*, 2018*b* ▸). Here we report the synthesis, structural characterization and Hirshfeld surface analysis of a related ligand that crystallizes as its di­methyl­sulfoxide monosolvate, C_21_H_12_N_4_O·(CH_3_)_2_SO.
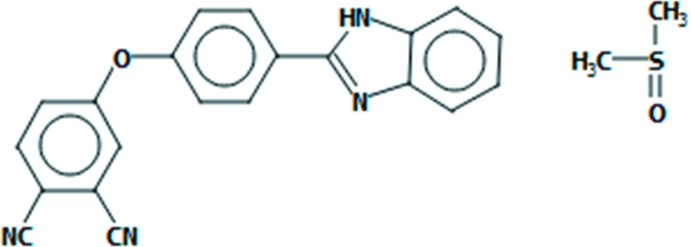


## Structural commentary   

**Figure 1:**
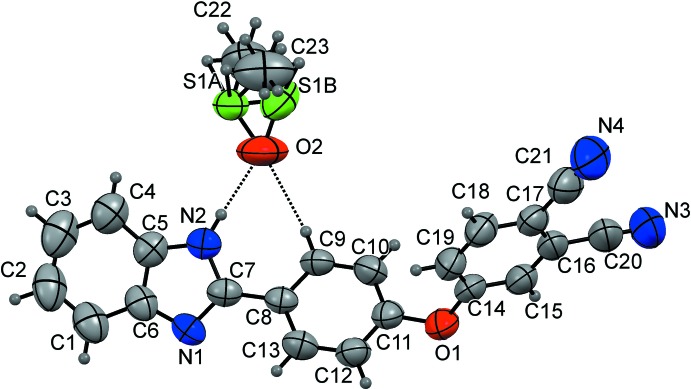
The mol­ecular structure of the title compound, showing the atom labelling. Displacement ellipsoids are drawn at the 50% probability level. Hydrogen bonds (Table 1 ▸) are shown as dashed lines.

The mol­ecular components of the title compound are shown in Fig. 1 ▸. The mol­ecular structure of the phthalo­nitrile derivative is constructed from three ring systems, *viz*. a central phen­oxy ring, a terminal phthalo­nitrile system and a terminal benzimidazole ring. The bond lengths of the cyano groups, 1.132 (6) and 1.137 (6) Å, for C21≡N4 and C20≡N3, respectively, conform well with literature values (Saraçoğlu *et al.*, 2011 ▸). The corresponding C—C≡N angles [179.4 (6) and 177.9 (7)°] are almost linear and are also in good agreement with literature values (Saraçoğlu *et al.*, 2011 ▸; Sen *et al.*, 2018*a* ▸). The C—C bond lengths of the phenyl rings are in the normal range of 1.356 (5)–1.395 (6) Å, *i.e.* characteristic of a delocalized system. The dihedral angle of 2.11 (1)° between the fused C1–C6 and C5/N2/C7/N1/C6 rings in the heterocycle indicate a minute deviation from planarity, whereas the attached C8–C13 ring is inclined by 20.7 (1)° to the C5/N2/C7/N1/C6 ring plane.

## Supra­molecular features   

**Figure 2:**
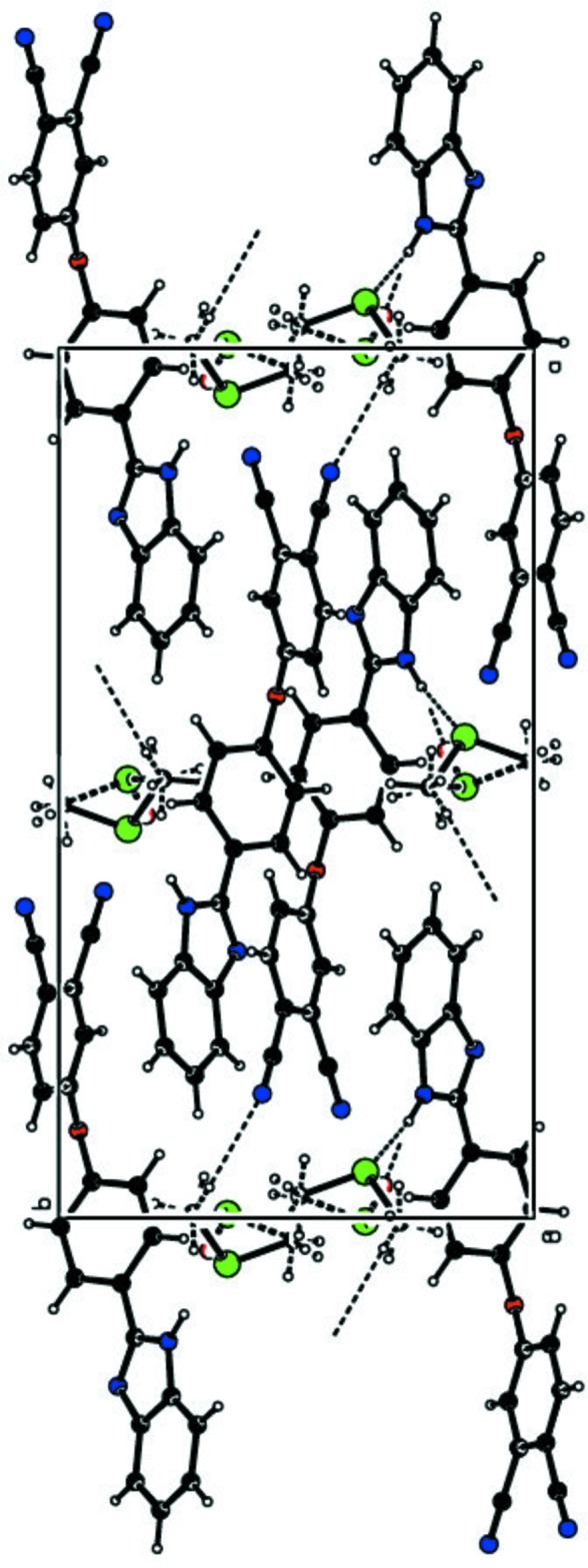
A view of the crystal packing of the title compound. Dashed lines denote the N2—H2⋯S1*A*, N2—H2⋯O2 and C23—H23*D*⋯N4 inter­molecular hydrogen-bonding inter­actions.

In the crystal structure, N2—H2⋯O2 and C9—H9⋯O2 inter­molecular hydrogen bonding inter­actions with an 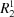
(7) graph-set motif are present, whereby the O2 atom acts as an acceptor in both cases (Fig. 1 ▸). There are also weak inter­molecular N2—H2⋯S1*A* inter­actions between the the N—H group of the imidazole ring and the disordered dimethyl sulfate solvent, and a C23—H23*D*⋯N4 inter­action between one of the methyl groups of the dimethyl sulfoxide solvent and one of the nitrile N atoms (Table 1 ▸, Fig. 2 ▸). These inter­actions lead to the formation of a three-dimensional supra­molecular network.

## Database survey   

A search of the Cambridge Structural database (CSD, version 5.40, update November 2018; Groom *et al.*, 2016 ▸) for the 4-[4-(1*H*-benzo[*d*]imidazole-2­yl)phen­oxy]phthalo­nitrile moiety revealed two hits. Distinctive bond lengths (N4≡C21, N3≡C20, C7—N2, C5—N2) in the title structure are the same within standard uncertainties as the corresponding bond lengths in the structures of 4-[4-(1*H*-benzimidazol-2-yl)phen­oxy]benzene-1,2-dicarbo­nitrile monohydrate (HIDHEK; Sen *et al.*, 2018*b* ▸) or 4-{4-[1-(prop-2-en-1-yl)-1*H*-benzimidazol-2-yl]phen­oxy}benzene-1,2-dicarbo­nitrile (RELBUI; Sen *et al.*, 2018*a* ▸). In these structures, the C—O bond lengths vary from 1.363–1.407 Å. In the title mol­ecule, the corresponding bond lengths are 1.367 (5) and 1.406 (4) Å, respectively. In all these structures, the mol­ecules are linked into chains by C—H⋯N hydrogen bonds.

## Hirshfeld surface analysis   

**Figure 3:**
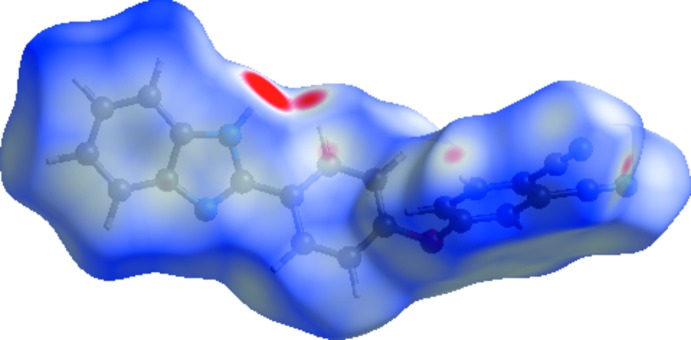
The Hirshfeld surface of the title compound mapped with *d*_norm_ in the range −0.6328 to 1.3784 a.u.

**Figure 4:**
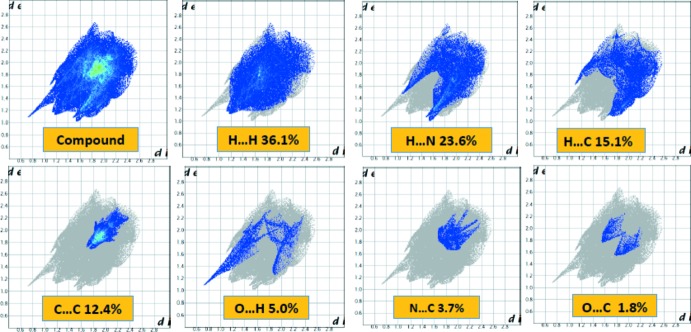
Two-dimensional fingerprint plots with a *d*_norm_ view of all inter­actions in the title compound, and subdivided into H⋯H (36.1%), N⋯H/H⋯N(23.6%), C⋯H/H⋯C (15.1%), C⋯C/C⋯C (12.4%), O⋯H/H⋯O (5.0%), C⋯N/N⋯C (3.7%), C⋯O/O⋯C (1.8%) and S⋯H/H⋯S (1.6%) contacts.

The Hirshfeld surface analysis (Spackman & Jayatilaka, 2009 ▸) and the associated two-dimensional fingerprint plots (McKinnon *et al.*, 2007 ▸) were performed with *CrystalExplorer17* (Turner *et al.*, 2017 ▸). The Hirshfeld surfaces were generated using a standard (high) surface resolution with the three-dimensional surfaces mapped over *d*_norm_ (Fig. 3 ▸). For the title mol­ecule, the H⋯H inter­actions appear in the middle of the scattered points in the fingerprint plots with a contribution to the overall Hirshfeld surface of 36.1% (Fig. 4 ▸). The contribution from the N⋯H/H⋯N contacts, corresponding to the C—H⋯N inter­actions, is represented by a pair of sharp spikes characteristic of a rather strong hydrogen-bonding inter­action (23.6%). The whole fingerprint region and all other inter­actions are displayed in Fig. 4 ▸. In particular, the O⋯H/H⋯O contacts indicate the presence of inter­molecular C—H⋯O and N—H⋯O inter­actions.

**Figure 5:**
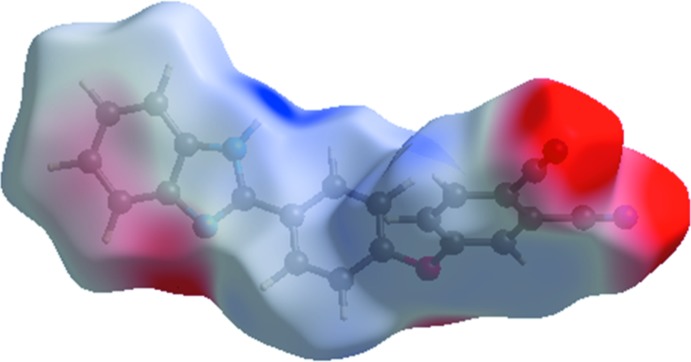
A view of the three-dimensional Hirshfeld surface of the title compound plotted over electrostatic potentials in the range −0.0893 to 0.1930 a.u.

A view of the mol­ecular electrostatic potential for the title compound, using the STO-3G basis set at the Hartree–Fock level of theory, is shown in Fig. 5 ▸. The N—H⋯N and C—H⋯N hydrogen-bond donor and acceptor groups are shown as blue and red areas around the atoms related with positive (hydrogen-bond donors) and negative (hydrogen-bond acceptors) electrostatic potentials, respectively.

## Synthesis and crystallization   

2-(4-Hy­droxy-phen­yl)-benzimidazole (1.2 g, 5.71 mmol), which was synthesized by the reaction of *o*-phenyl­enedi­amine and 4-hy­droxy­benzaldehyde, and 4-nitro­phthalo­nitrile (0.989 g, 5.71 mmol) were dissolved in DMF (15 ml) and degassed by argon in a dual-bank vacuum-gas manifold system. After stirring for 15 min, finely ground anhydrous K_2_CO_3_ (0.790 g, 5.71 mmol) was added portion-wise over 2 h under stirring. The suspension solution was maintained at 333 K for 24 h. After completion of the reaction, the crude product was precipitated by pouring into ice–water. The precipitate was collected by filtration, washed with hot water, ethanol, diethyl ether and was finally dried *in vacuo*. The desired compound was obtained in sufficient purity. The obtained spectroscopic data are accordance with the literature (Khan *et al.*, 2009 ▸). Single crystals for structure analysis were obtained from slow evaporation of a DMSO solution.

## Refinement   

Crystal data, data collection and structure refinement details are summarized in Table 2 ▸. H atoms were positioned geometrically and allowed to ride on their parent atoms, with C—H = 0.93 Å for aromatic groups, with N—H = 0.86 Å for the imidazole moiety and with 0.96 Å for methyl groups. *U*_iso_(H) values were constrained to 1.2–1.5 U_eq_ of their carrier atoms. The sulfur atom of the di­methyl­sulfate solvent is disordered over two sites (S1*A* and S1*B*), with an occupancy ratio of 0.623 (5):0.377 (5).

## Supplementary Material

Crystal structure: contains datablock(s) I. DOI: 10.1107/S2056989019006510/wm5496sup1.cif

Structure factors: contains datablock(s) I. DOI: 10.1107/S2056989019006510/wm5496Isup2.hkl

Supporting information file. DOI: 10.1107/S2056989019006510/wm5496Isup3.cml

CCDC reference: 1846754

Additional supporting information:  crystallographic information; 3D view; checkCIF report

## Figures and Tables

**Table 1:** Hydrogen-bond geometry (Å, °)

*D*—H⋯*A*	*D*—H	H⋯*A*	*D*⋯*A*	*D*—H⋯*A*
N2—H2⋯O2	0.86	1.94	2.794 (5)	172
N2—H2⋯S1*A*	0.86	2.83	3.614 (4)	152
C9—H9⋯O2	0.93	2.40	3.175 (5)	141
C23—H23*D*⋯N4^i^	0.96	2.63	3.500 (9)	151

Symmetry code: (i) 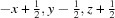.

**Table 2:** Experimental details

Crystal data	
Chemical formula	C_21_H_12_N_4_O·C_2_H_6_OS
*M*_r_	414.47
Crystal system, space group	Orthorhombic, *P* *n* *a*2_1_
Temperature (K)	296
*a*, *b*, *c* (Å)	20.9154 (11), 11.4208 (6), 8.8938 (6)
*V* (Å^3^)	2124.5 (2)
*Z*	4
Radiation type	Mo *K*α
μ (mm^−1^)	0.18
Crystal size (mm)	0.65 × 0.56 × 0.47

Data collection	
Diffractometer	Stoe IPDS 2
Absorption correction	Integration (*X-RED32*; Stoe & Cie, 2002 ▸)
*T*_min_, *T*_max_	0.966, 0.977
No. of measured, independent and observed [*I* > 2σ(*I*)] reflections	15225, 4660, 2281
*R*_int_	0.058
(sin θ/λ)_max_ (Å^−1^)	0.641

Refinement	
*R*[*F* ^2^ > 2σ(*F* ^2^)], *wR*(*F* ^2^), *S*	0.042, 0.098, 0.83
No. of reflections	4660
No. of parameters	281
No. of restraints	1
H-atom treatment	H-atom parameters constrained
Δρ_max_, Δρ_min_ (e Å^−3^)	0.20, −0.12
Absolute structure	Flack *x* determined using 771 quotients [(*I* ^+^)−(*I* ^−^)]/[(*I* ^+^)+(*I* ^−^)] (Parsons *et al.*, 2013 ▸)
Absolute structure parameter	−0.02 (8)

Computer programs: *X-AREA* and *X-RED32* (Stoe & Cie, 2002 ▸), *SHELXT2018* (Sheldrick, 2015*a* ▸), *SHELXL2018* (Sheldrick, 2015*b* ▸), *ORTEP-3 for Windows* and *WinGX* (Farrugia, 2012 ▸), *Mercury* (Macrae *et al.*, 2006 ▸) and *PLATON* (Spek, 2009 ▸).

## supplementary crystallographic information

### Crystal data

**Table:**

C_21_H_12_N_4_O·C_2_H_6_OS	*D*_x_ = 1.296 Mg m^−^^3^
*M**_r_* = 414.47	Mo *K*α radiation, λ = 0.71073 Å
Orthorhombic, *P**n**a*2_1_	Cell parameters from 9474 reflections
*a* = 20.9154 (11) Å	θ = 1.8–27.0°
*b* = 11.4208 (6) Å	µ = 0.18 mm^−^^1^
*c* = 8.8938 (6) Å	*T* = 296 K
*V* = 2124.5 (2) Å^3^	Prism, yellow
*Z* = 4	0.65 × 0.56 × 0.47 mm
*F*(000) = 864	

### Data collection

**Table:**

Stoe IPDS 2 diffractometer	4660 independent reflections
Radiation source: sealed X-ray tube, 12 x 0.4 mm long-fine focus	2281 reflections with *I* > 2σ(*I*)
Plane graphite monochromator	*R*_int_ = 0.058
Detector resolution: 6.67 pixels mm^-1^	θ_max_ = 27.1°, θ_min_ = 2.0°
rotation method scans	*h* = −26→22
Absorption correction: integration (X-RED32; Stoe & Cie, 2002)	*k* = −14→14
*T*_min_ = 0.966, *T*_max_ = 0.977	*l* = −11→11
15225 measured reflections	

### Refinement

**Table:**

Refinement on *F*^2^	Hydrogen site location: inferred from neighbouring sites
Least-squares matrix: full	H-atom parameters constrained
*R*[*F*^2^ > 2σ(*F*^2^)] = 0.042	*w* = 1/[σ^2^(*F*_o_^2^) + (0.0409*P*)^2^] where *P* = (*F*_o_^2^ + 2*F*_c_^2^)/3
*wR*(*F*^2^) = 0.098	(Δ/σ)_max_ < 0.001
*S* = 0.83	Δρ_max_ = 0.20 e Å^−^^3^
4660 reflections	Δρ_min_ = −0.12 e Å^−^^3^
281 parameters	Absolute structure: Flack *x* determined using 771 quotients [(*I*^+^)-(*I*^-^)]/[(*I*^+^)+(*I*^-^)] (Parsons *et al.*, 2013)
1 restraint	Absolute structure parameter: −0.02 (8)

### Special details

**Table:**

Geometry. All esds (except the esd in the dihedral angle between two l.s. planes) are estimated using the full covariance matrix. The cell esds are taken into account individually in the estimation of esds in distances, angles and torsion angles; correlations between esds in cell parameters are only used when they are defined by crystal symmetry. An approximate (isotropic) treatment of cell esds is used for estimating esds involving l.s. planes.

### Fractional atomic coordinates and isotropic or equivalent isotropic displacement parameters (Å^2^)

**Table:**

	*x*	*y*	*z*	*U*_iso_*/*U*_eq_	Occ. (<1)
S1A	0.55296 (11)	0.14556 (16)	0.7814 (2)	0.0869 (9)	0.623 (5)
S1B	0.4957 (2)	0.1452 (4)	0.7420 (4)	0.114 (2)	0.377 (5)
O1	0.39958 (15)	0.4586 (3)	0.0943 (3)	0.0925 (8)	
O2	0.53727 (19)	0.1838 (3)	0.6323 (4)	0.1425 (15)	
N1	0.69349 (16)	0.3756 (2)	0.2997 (4)	0.0751 (8)	
N2	0.64212 (17)	0.2699 (2)	0.4721 (3)	0.0720 (8)	
H2	0.611036	0.236598	0.518771	0.086*	
N3	0.1249 (2)	0.4056 (4)	0.1258 (7)	0.1429 (19)	
N4	0.1428 (2)	0.5716 (5)	0.5187 (7)	0.156 (2)	
C1	0.8034 (2)	0.3391 (4)	0.4030 (6)	0.0968 (13)	
H1	0.824993	0.380986	0.329242	0.116*	
C2	0.8361 (3)	0.2860 (5)	0.5198 (7)	0.1100 (17)	
H2A	0.880352	0.293117	0.526112	0.132*	
C3	0.8032 (3)	0.2223 (5)	0.6272 (7)	0.1098 (16)	
H3	0.826349	0.187242	0.704234	0.132*	
C4	0.7380 (3)	0.2085 (4)	0.6256 (6)	0.0957 (13)	
H4	0.716824	0.165147	0.698781	0.115*	
C5	0.7054 (2)	0.2630 (3)	0.5081 (4)	0.0724 (10)	
C6	0.7375 (2)	0.3280 (3)	0.3992 (5)	0.0748 (10)	
C7	0.6375 (2)	0.3395 (3)	0.3484 (4)	0.0652 (10)	
C8	0.57530 (16)	0.3683 (3)	0.2821 (4)	0.0605 (8)	
C9	0.52110 (19)	0.3027 (3)	0.3118 (4)	0.0679 (10)	
H9	0.523936	0.237162	0.373482	0.081*	
C10	0.46302 (19)	0.3338 (3)	0.2509 (4)	0.0785 (11)	
H10	0.427035	0.288323	0.270114	0.094*	
C11	0.45811 (19)	0.4303 (4)	0.1630 (4)	0.0716 (10)	
C12	0.5104 (2)	0.4970 (4)	0.1317 (5)	0.0783 (11)	
H12	0.506725	0.562819	0.070782	0.094*	
C13	0.5690 (2)	0.4659 (3)	0.1912 (4)	0.0752 (11)	
H13	0.604801	0.511332	0.169824	0.090*	
C14	0.3486 (2)	0.4793 (3)	0.1868 (5)	0.0734 (11)	
C15	0.2890 (2)	0.4466 (3)	0.1358 (5)	0.0804 (11)	
H15	0.284932	0.408992	0.043566	0.096*	
C16	0.2358 (2)	0.4694 (4)	0.2210 (6)	0.0827 (12)	
C17	0.2414 (2)	0.5253 (4)	0.3584 (5)	0.0845 (12)	
C18	0.3018 (2)	0.5574 (4)	0.4094 (5)	0.0890 (12)	
H18	0.306198	0.594294	0.502000	0.107*	
C19	0.3549 (2)	0.5347 (4)	0.3231 (5)	0.0820 (11)	
H19	0.395119	0.556988	0.357067	0.098*	
C20	0.1739 (3)	0.4332 (4)	0.1682 (6)	0.1069 (17)	
C21	0.1857 (3)	0.5498 (5)	0.4466 (6)	0.1113 (17)	
C22	0.5253 (2)	0.0068 (4)	0.8099 (6)	0.1185 (17)	
H22A	0.536012	−0.018040	0.909946	0.178*	0.623 (5)
H22B	0.479713	0.005567	0.797600	0.178*	0.623 (5)
H22C	0.544633	−0.045215	0.738362	0.178*	0.623 (5)
H22D	0.497256	−0.022369	0.886737	0.178*	0.377 (5)
H22E	0.527035	−0.048198	0.728417	0.178*	0.377 (5)
H22F	0.567387	0.017228	0.850914	0.178*	0.377 (5)
C23	0.4951 (4)	0.2180 (5)	0.8988 (7)	0.151 (2)	
H23A	0.502326	0.196930	1.001908	0.227*	0.623 (5)
H23B	0.499347	0.301270	0.887593	0.227*	0.623 (5)
H23C	0.452845	0.194538	0.869547	0.227*	0.623 (5)
H23D	0.464660	0.183633	0.966396	0.227*	0.377 (5)
H23E	0.536893	0.215626	0.943443	0.227*	0.377 (5)
H23F	0.483454	0.297886	0.879270	0.227*	0.377 (5)

### Atomic displacement parameters (Å^2^)

**Table:**

	*U*^11^	*U*^22^	*U*^33^	*U*^12^	*U*^13^	*U*^23^
S1A	0.0892 (19)	0.1040 (13)	0.0676 (11)	−0.0240 (11)	−0.0011 (11)	0.0104 (10)
S1B	0.124 (5)	0.131 (3)	0.087 (3)	0.016 (3)	−0.017 (2)	0.002 (2)
O1	0.081 (2)	0.135 (2)	0.0614 (15)	0.0085 (18)	0.0017 (16)	0.0154 (17)
O2	0.159 (4)	0.159 (3)	0.109 (3)	0.021 (3)	0.056 (3)	0.056 (2)
N1	0.068 (2)	0.0793 (19)	0.078 (2)	−0.0039 (17)	0.0160 (19)	−0.0036 (19)
N2	0.078 (2)	0.0676 (19)	0.070 (2)	−0.0024 (17)	0.0048 (19)	−0.0014 (16)
N3	0.097 (3)	0.166 (4)	0.165 (4)	−0.038 (3)	−0.028 (3)	0.051 (4)
N4	0.102 (4)	0.230 (6)	0.135 (4)	0.061 (4)	0.025 (3)	0.029 (4)
C1	0.080 (3)	0.110 (3)	0.100 (3)	0.005 (3)	0.014 (3)	−0.028 (3)
C2	0.079 (3)	0.138 (5)	0.113 (4)	0.030 (3)	−0.003 (4)	−0.042 (4)
C3	0.111 (5)	0.112 (4)	0.106 (4)	0.052 (3)	−0.008 (4)	−0.028 (3)
C4	0.103 (4)	0.083 (3)	0.101 (3)	0.023 (3)	−0.001 (3)	−0.010 (3)
C5	0.084 (3)	0.062 (2)	0.072 (3)	0.014 (2)	0.001 (3)	−0.011 (2)
C6	0.068 (3)	0.078 (3)	0.078 (3)	0.006 (2)	0.009 (2)	−0.019 (2)
C7	0.079 (3)	0.0538 (19)	0.063 (2)	−0.001 (2)	0.011 (2)	−0.0006 (19)
C8	0.070 (2)	0.0558 (19)	0.0556 (19)	−0.0026 (18)	0.010 (2)	−0.0016 (19)
C9	0.079 (3)	0.064 (2)	0.061 (2)	−0.009 (2)	0.008 (2)	0.0118 (18)
C10	0.075 (3)	0.085 (3)	0.076 (3)	−0.013 (2)	0.008 (2)	0.010 (2)
C11	0.071 (3)	0.087 (3)	0.057 (2)	0.007 (2)	0.006 (2)	0.010 (2)
C12	0.084 (3)	0.076 (3)	0.075 (3)	0.002 (2)	0.007 (2)	0.020 (2)
C13	0.081 (3)	0.067 (2)	0.077 (3)	−0.008 (2)	0.011 (2)	0.007 (2)
C14	0.074 (3)	0.085 (3)	0.062 (2)	0.003 (2)	−0.004 (2)	0.022 (2)
C15	0.084 (3)	0.086 (3)	0.071 (3)	−0.005 (2)	−0.013 (2)	0.020 (2)
C16	0.067 (3)	0.087 (3)	0.094 (3)	−0.004 (2)	−0.007 (3)	0.037 (3)
C17	0.075 (3)	0.099 (3)	0.079 (3)	0.016 (2)	0.000 (3)	0.029 (3)
C18	0.088 (4)	0.106 (3)	0.073 (3)	0.021 (3)	−0.007 (3)	0.007 (3)
C19	0.073 (3)	0.103 (3)	0.071 (3)	0.004 (2)	−0.009 (2)	0.007 (2)
C20	0.085 (3)	0.117 (4)	0.119 (4)	−0.012 (3)	−0.012 (3)	0.050 (3)
C21	0.089 (4)	0.139 (4)	0.106 (4)	0.034 (3)	0.008 (3)	0.034 (3)
C22	0.150 (5)	0.096 (3)	0.110 (4)	−0.009 (3)	0.006 (3)	0.021 (3)
C23	0.231 (7)	0.101 (4)	0.122 (4)	0.003 (4)	0.046 (5)	−0.007 (4)

### Geometric parameters (Å, º)

**Table:**

S1A—O2	1.434 (4)	C10—C11	1.356 (5)
S1A—C22	1.706 (5)	C10—H10	0.9300
S1A—C23	1.800 (6)	C11—C12	1.362 (5)
S1B—O2	1.379 (5)	C12—C13	1.381 (5)
S1B—C23	1.624 (7)	C12—H12	0.9300
S1B—C22	1.801 (6)	C13—H13	0.9300
O1—C14	1.367 (5)	C14—C19	1.374 (5)
O1—C11	1.406 (4)	C14—C15	1.378 (5)
N1—C7	1.314 (4)	C15—C16	1.372 (6)
N1—C6	1.388 (5)	C15—H15	0.9300
N2—C7	1.361 (4)	C16—C17	1.384 (6)
N2—C5	1.365 (5)	C16—C20	1.437 (7)
N2—H2	0.8600	C17—C18	1.390 (6)
N3—C20	1.137 (6)	C17—C21	1.433 (7)
N4—C21	1.132 (6)	C18—C19	1.375 (5)
C1—C6	1.384 (6)	C18—H18	0.9300
C1—C2	1.384 (7)	C19—H19	0.9300
C1—H1	0.9300	C22—H22A	0.9600
C2—C3	1.384 (7)	C22—H22B	0.9600
C2—H2A	0.9300	C22—H22C	0.9600
C3—C4	1.372 (7)	C22—H22D	0.9600
C3—H3	0.9300	C22—H22E	0.9600
C4—C5	1.395 (6)	C22—H22F	0.9600
C4—H4	0.9300	C23—H23A	0.9600
C5—C6	1.392 (5)	C23—H23B	0.9600
C7—C8	1.466 (5)	C23—H23C	0.9600
C8—C9	1.384 (5)	C23—H23D	0.9600
C8—C13	1.384 (5)	C23—H23E	0.9600
C9—C10	1.376 (5)	C23—H23F	0.9600
C9—H9	0.9300		
			
O2—S1A—C22	110.0 (3)	C12—C13—C8	121.0 (4)
O2—S1A—C23	104.1 (3)	C12—C13—H13	119.5
C22—S1A—C23	96.5 (3)	C8—C13—H13	119.5
O2—S1B—C23	116.7 (4)	O1—C14—C19	122.5 (4)
O2—S1B—C22	107.6 (4)	O1—C14—C15	117.4 (4)
C23—S1B—C22	99.4 (3)	C19—C14—C15	120.1 (4)
C14—O1—C11	117.2 (3)	C16—C15—C14	120.1 (4)
C7—N1—C6	104.9 (3)	C16—C15—H15	120.0
C7—N2—C5	106.9 (3)	C14—C15—H15	120.0
C7—N2—H2	126.5	C15—C16—C17	120.3 (4)
C5—N2—H2	126.5	C15—C16—C20	119.8 (5)
C6—C1—C2	118.1 (5)	C17—C16—C20	119.9 (5)
C6—C1—H1	120.9	C16—C17—C18	119.2 (5)
C2—C1—H1	120.9	C16—C17—C21	120.3 (5)
C1—C2—C3	120.1 (5)	C18—C17—C21	120.6 (5)
C1—C2—H2A	120.0	C19—C18—C17	120.1 (4)
C3—C2—H2A	120.0	C19—C18—H18	119.9
C4—C3—C2	123.3 (5)	C17—C18—H18	119.9
C4—C3—H3	118.4	C14—C19—C18	120.2 (4)
C2—C3—H3	118.4	C14—C19—H19	119.9
C3—C4—C5	116.2 (5)	C18—C19—H19	119.9
C3—C4—H4	121.9	N3—C20—C16	179.4 (6)
C5—C4—H4	121.9	N4—C21—C17	177.9 (7)
N2—C5—C6	105.9 (4)	S1A—C22—H22A	109.5
N2—C5—C4	132.5 (4)	S1A—C22—H22B	109.5
C6—C5—C4	121.6 (5)	H22A—C22—H22B	109.5
C1—C6—N1	129.8 (4)	S1A—C22—H22C	109.5
C1—C6—C5	120.7 (4)	H22A—C22—H22C	109.5
N1—C6—C5	109.4 (4)	H22B—C22—H22C	109.5
N1—C7—N2	112.8 (4)	S1B—C22—H22D	109.5
N1—C7—C8	126.0 (3)	S1B—C22—H22E	109.5
N2—C7—C8	121.2 (3)	H22D—C22—H22E	109.5
C9—C8—C13	118.0 (4)	S1B—C22—H22F	109.5
C9—C8—C7	122.0 (3)	H22D—C22—H22F	109.5
C13—C8—C7	120.0 (3)	H22E—C22—H22F	109.5
C10—C9—C8	120.6 (4)	S1A—C23—H23A	109.5
C10—C9—H9	119.7	S1A—C23—H23B	109.5
C8—C9—H9	119.7	H23A—C23—H23B	109.5
C11—C10—C9	120.2 (4)	S1A—C23—H23C	109.5
C11—C10—H10	119.9	H23A—C23—H23C	109.5
C9—C10—H10	119.9	H23B—C23—H23C	109.5
C10—C11—C12	120.8 (4)	S1B—C23—H23D	109.5
C10—C11—O1	120.3 (4)	S1B—C23—H23E	109.5
C12—C11—O1	118.8 (4)	H23D—C23—H23E	109.5
C11—C12—C13	119.4 (4)	S1B—C23—H23F	109.5
C11—C12—H12	120.3	H23D—C23—H23F	109.5
C13—C12—H12	120.3	H23E—C23—H23F	109.5
			
C6—C1—C2—C3	1.1 (7)	C8—C9—C10—C11	−1.2 (6)
C1—C2—C3—C4	−0.3 (7)	C9—C10—C11—C12	1.0 (6)
C2—C3—C4—C5	−0.2 (7)	C9—C10—C11—O1	176.6 (3)
C7—N2—C5—C6	−1.6 (4)	C14—O1—C11—C10	60.5 (5)
C7—N2—C5—C4	177.2 (4)	C14—O1—C11—C12	−123.7 (4)
C3—C4—C5—N2	−178.8 (4)	C10—C11—C12—C13	−0.4 (6)
C3—C4—C5—C6	−0.1 (6)	O1—C11—C12—C13	−176.1 (4)
C2—C1—C6—N1	177.1 (4)	C11—C12—C13—C8	−0.1 (6)
C2—C1—C6—C5	−1.4 (6)	C9—C8—C13—C12	−0.1 (5)
C7—N1—C6—C1	−178.8 (4)	C7—C8—C13—C12	−177.8 (4)
C7—N1—C6—C5	−0.2 (4)	C11—O1—C14—C19	36.2 (5)
N2—C5—C6—C1	179.9 (3)	C11—O1—C14—C15	−146.4 (3)
C4—C5—C6—C1	0.9 (6)	O1—C14—C15—C16	−177.3 (3)
N2—C5—C6—N1	1.1 (4)	C19—C14—C15—C16	0.1 (6)
C4—C5—C6—N1	−177.9 (3)	C14—C15—C16—C17	0.0 (6)
C6—N1—C7—N2	−0.9 (4)	C14—C15—C16—C20	−178.9 (4)
C6—N1—C7—C8	178.9 (3)	C15—C16—C17—C18	−0.3 (6)
C5—N2—C7—N1	1.6 (4)	C20—C16—C17—C18	178.6 (4)
C5—N2—C7—C8	−178.1 (3)	C15—C16—C17—C21	179.5 (4)
N1—C7—C8—C9	161.5 (3)	C20—C16—C17—C21	−1.6 (6)
N2—C7—C8—C9	−18.8 (5)	C16—C17—C18—C19	0.6 (6)
N1—C7—C8—C13	−20.9 (5)	C21—C17—C18—C19	−179.2 (4)
N2—C7—C8—C13	158.9 (3)	O1—C14—C19—C18	177.5 (4)
C13—C8—C9—C10	0.7 (5)	C15—C14—C19—C18	0.2 (5)
C7—C8—C9—C10	178.3 (3)	C17—C18—C19—C14	−0.6 (6)

### Hydrogen-bond geometry (Å, º)

**Table:**

*D*—H···*A*	*D*—H	H···*A*	*D*···*A*	*D*—H···*A*
N2—H2···O2	0.86	1.94	2.794 (5)	172
N2—H2···S1*A*	0.86	2.83	3.614 (4)	152
C9—H9···O2	0.93	2.40	3.175 (5)	141
C23—H23*D*···N4^i^	0.96	2.63	3.500 (9)	151

Symmetry code: (i) −*x*+1/2, *y*−1/2, *z*+1/2.

## References

[bb1] El Rashedy, A. A. & Aboul-Enein, H. Y. (2013). *Mini Rev. Med. Chem.* **13**, 399–407. 10.2174/138955713804999847 23190032

[bb2] Farrugia, L. J. (2012). *J. Appl. Cryst.* **45**, 849–854.

[bb3] Gaba, M., Singh, S. & Mohan, C. (2014). *Eur. J. Med. Chem.* **76**, 494–505. 10.1016/j.ejmech.2014.01.030 24602792

[bb4] Groom, C. R., Bruno, I. J., Lightfoot, M. P. & Ward, S. C. (2016). *Acta Cryst.* B**72**, 171–179. 10.1107/S2052520616003954 PMC4822653 27048719

[bb5] Kathiravan, M. K., Salake, A. B., Chothe, A. S., Dudhe, P. B., Watode, R. P., Mukta, M. S. & Gadhwe, S. (2012). *Bioorg. Med. Chem.* **20**, 5678–5698. 10.1016/j.bmc.2012.04.045 22902032

[bb6] Khan, A. T., Parvin, T. & Choudhury, L. H. (2009). *Synth. Commun.* **39**, 2339–2346.

[bb7] Macrae, C. F., Edgington, P. R., McCabe, P., Pidcock, E., Shields, G. P., Taylor, R., Towler, M. & van de Streek, J. (2006). *J. Appl. Cryst.* **39**, 453–457.

[bb8] Martínez-Díaz, M. V., Ince, M. & Torres, T. (2011). *Monatsh. Chem.* **142**, 699–707.

[bb9] McKinnon, J. J., Jayatilaka, D. & Spackman, M. A. (2007). *Chem. Commun.*, pp. 3814–3816. 10.1039/b704980c 18217656

[bb10] Parsons, S., Flack, H. D. & Wagner, T. (2013). *Acta Cryst.* B**69**, 249–259. 10.1107/S2052519213010014 PMC3661305 23719469

[bb11] Saraçoğlu, H., Güntepe, F., Yüksektepe, Ç. N. & Saydam, S. (2011). *Mol. Cryst. Liq. Cryst.* **537**, 111–127.

[bb12] Sen, P., Atmaca, G. Y., Erdogmus, A., Kanmazalp, S. D., Dege, N. & Yildiz, S. Z. (2018*b*). *J. Lumin.* **194**, 123–130.

[bb13] Sen, P., Kansiz, S., Dege, N., Iskenderov, T. S. & Yildiz, S. Z. (2018*a*). *Acta Cryst.* E**74**, 994–997. 10.1107/S2056989018008745 PMC6038632 30002901

[bb14] Sheldrick, G. M. (2015*a*). *Acta Cryst.* A**71**, 3–8.

[bb15] Sheldrick, G. M. (2015*b*). *Acta Cryst.* C**71**, 3–8.

[bb16] Spackman, M. A. & Jayatilaka, D. (2009). *CrystEngComm*, **11**, 19–32.

[bb17] Spek, A. L. (2009). *Acta Cryst.* D**65**, 148–155. 10.1107/S090744490804362X PMC2631630 19171970

[bb18] Srestha, N., Banerjee, J. & Srivastava, S. (2014). *IOSR J. Pharma* **4**, 28–41.

[bb19] Stoe & Cie (2002). *X-AREA* and *X-RED32*. Stoe & Cie GmbH, Darmstadt, Germany.

[bb20] Torre, G. de la, Vázquez, P., Agulló-López, F. & Torres, T. (2004). *Chem. Rev.* **104**, 3723–3750. 10.1021/cr030206t 15352778

[bb21] Turner, M. J., McKinnon, J. J., Wolff, S. K., Grimwood, D. J., Spackman, P. R., Jayatilaka, D. & Spackman, M. A. (2017). *CrystalExplorer17.* University of Western Australia. http://hirshfeldsurface.net

